# Exploring Replicative Senescence and Oxidative Stress-Induced Remodelling of Mitochondrial-Associated Membranes in Human Skin Fibroblasts

**DOI:** 10.3390/biom16050704

**Published:** 2026-05-11

**Authors:** Anne-Laure Bulteau, Gallic Beauchef, Stéphanie Chanon, Aurélie Vieille-Marchis, Julien Chlasta, Gaël Runel, Juliette Sage, Tanesha Naiken, Lauren Sobilo, Elodie Bossard, Lorene Gourguillon, Carine Nizard, Karl Pays, Laurence Canaple, Beatrice Morio

**Affiliations:** 1 LVMH Recherche, 185 Avenue de Verdun, 45800 Saint Jean de Braye, France; gbeauchef@research.lvmh-pc.com (G.B.); jsage@research.lvmh-pc.com (J.S.); tnaiken@research.lvmh-pc.com (T.N.); lsobilo@research.lvmh-pc.com (L.S.); ebossard@research.lvmh-pc.com (E.B.); lgourguillon@research.lvmh-pc.com (L.G.); cnizard@research.lvmh-pc.com (C.N.); kpays@research.lvmh-pc.com (K.P.); 2 Laboratoire CarMeN, UMR INSERM U1060/INRAe U1397, Université Claude Bernard Lyon1, 69310 Pierre-Bénite, France; stephanie.chanon@univ-lyon1.fr (S.C.); aurelie.vieille-marchiset@inserm.fr (A.V.-M.); 3 BIOMECA, 69008 Lyon, France; julien.chlasta@bio-meca.com (J.C.); gael.runel@bio-meca.com (G.R.); 4 SFR Biosciences, AniRA-ImmOs, CNRS UAR 3444, Inserm US8, UCBL, ENS de Lyon, 69007 Lyon, France; laurence.canaple@ens-lyon.fr

**Keywords:** skin ageing, mitochondria-ER contact sites, oxidative stress, intra-mitochondrial calcium, Bioenergetic Health Index, cytoskeleton physical properties

## Abstract

(1) Background: Calcium transfer between the endoplasmic reticulum (ER) and mitochondria through the IP3R–VDAC1 complex at mitochondria-associated ER membranes (MAMs) is essential for cellular homeostasis. Alterations in this signalling axis have been implicated in ageing and cellular senescence. (2) Methods: We developed an in vitro human dermal fibroblast (HDF) model combining replicative senescence and acute oxidative stress to investigate the role of ER–mitochondria coupling in skin ageing and to enable biomolecule screening. (3) Results: In situ proximity ligation assays revealed that replicative senescence significantly increased the number of VDAC1/IP3R complexes per cell (+85% and +72%, *p* < 0.01), together with elevated cellular reactive oxygen species (+47% and +74%, *p* < 0.05). Consistently, acute oxidative stress (50 µM t-BHP, 30 min) rapidly increased VDAC1/IP3R complexes (+48%, *p* < 0.001) and intra-mitochondrial calcium levels (+19%, *p* < 0.001). These effects persisted for 24 h post-treatment and were associated with impaired mitochondrial function (−27% in the Bioenergetic Health Index, *p* < 0.05). We also established a flexibility index capturing both acute and long-term adaptations and detecting the protective effects of an orchid extract. (4) Conclusions: ER–mitochondria coupling disruption via the IP3R–VDAC1 complex may contribute to oxidative stress-induced senescence and represent a key mechanism in extrinsic skin ageing.

## 1. Introduction

Membrane contact sites (MCSs) are regions where the membranes of distinct organelles, or the membrane of an organelle and the plasma membrane, are closely apposed without undergoing fusion [1,2,3]. These highly dynamic structures serve multiple specific functions and exhibit a distinct protein and lipid composition. MCSs are stabilized by tethering proteins that maintain the two membranes in proximity, typically separated by a 10 to 80 nm gap. While MCSs can form between various cellular structures, a significant proportion involve the endoplasmic reticulum (ER), the largest membrane-bound organelle whose membrane contributes to more than half of the total cellular membranes [2].

Among the various MCSs, those formed between the ER and mitochondria are the most extensively characterized [4,5]. These mitochondria-associated ER membranes (MAMs), also referred to as mitochondria–ER contact sites (MERCs), span 5 to 20% of the mitochondrial outer membrane [6]. In addition to protein tethers, more than a thousand proteins are associated with MAMs, with some being only transiently present, others exhibiting a higher proportion in MAMs than in other cellular compartments, and some being unique to MAMs [7,8]. While MAM protein composition is highly heterogeneous across cell types, it includes ion channels, transport proteins, enzymes, and signalling molecules [4]. Consequently, MAMs are crucial for numerous cellular processes, including Ca^2+^ transfer, lipid trafficking and metabolism, mitochondrial dynamics, reactive oxygen species (ROS) modulation, apoptosis, and autophagy [4,5,9,10,11,12]. They are hubs for inter-organelle communication, regulating organelle functions and maintaining metabolic homeostasis.

Multiple lines of evidence indicate that alterations in the quality and quantity of MAMs are a hallmark of several age-related diseases [10,13,14]. Pathological conditions such as diabetes, cardiovascular diseases, neurodegenerative disorders, and cancer have all been linked to MAM disruption. Ageing also impacts MAMs, with several factors converging to disrupt their structure and function over time, contributing to Ca^2+^ imbalance and triggering cellular stress and dysfunction [15,16,17].

The precise role of MAM alterations in age-related diseases and senescence remains to be fully elucidated. Nevertheless, these alterations may further promote cellular senescence and ageing, suggesting that MAMs could contribute to the regulation of ageing [13,14]. Regardless of their exact function, it is becoming increasingly evident that MAM alterations represent an early marker of cellular dysfunction. Monitoring these alterations could enable the tracking of cellular senescence and potentially lead to the development of novel preventive strategies.

Despite technological advancements, MAMs remain challenging to observe and isolate unequivocally, complicating efforts to assess their modulation by various factors [3,18]. Initially identified by transmission electron microscopy (TEM) [19], this technique is poorly suited for studying MAMs in precompetitive settings requiring high throughput due to its time-consuming nature. Subcellular fractionation and density gradient centrifugation have proven to be valuable techniques despite the risk of contamination and the potential loss of key components [20]. Various microscopy techniques, primarily confocal microscopy, took advantage of proximity labelling or proximity-driven signal generation [21]. Several studies have validated the consistency of MAM integrity evaluated using either a transmission electron microscope (TEM) or the quantification of the close contact between the ER IP3R receptor and the mitochondrial VDAC1 channel, a key complex involved in calcium transfer from the ER to the mitochondria that requires MAM integrity to function [22,23,24,25]. The latter approach can use the in situ proximity ligation assay (PLA), which detects proteins located in close proximity (≤40 nm) using pairs of antibodies coupled to complementary DNA oligonucleotides [26].

Oxidative stress is a significant contributing factor to replicative senescence in vitro [27,28]. It plays a dual role in both intrinsic and extrinsic ageing processes, functioning simultaneously as a signalling molecule and as a catalyst for cellular damage. Considering that oxidative stress represents a key characteristic of environmental challenges encountered by skin tissue, we sought to establish an acute oxidative stress model that recapitulates key features of replication-induced senescence and enables efficient biomolecule screening. To this end, we characterized human dermal fibroblasts (HDFs) that had undergone replicative senescence. We then established a model on young HDFs exposed to acute treatment with tert-butyl hydroperoxide (t-BHP) that mimics these alterations [29]. Finally, we tested the protective effect of an orchid extract *Gastrodia elata*, whose main constituent, gastrodin, has demonstrated protective effects on cardiomyocytes against oxidative injury by promoting the nuclear translocation of Nrf2, regulating mitochondrial dynamics, and preserving mitochondrial structure and function [30]. To that purpose, the number of VDAC1/IP3R complexes per cell, intra-mitochondrial calcium, mitochondrial respiration, plasma membrane fluidity, and cytoskeleton mechanical characteristics were assessed. Finally, we also derived a flexibility index of MAM alterations to facilitate the screening of biomolecules.

## 2. Materials and Methods

### 2.1. Skin Tissue Procurement

The primary skin cell cultures were established individually from an abdominal skin biopsy obtained from a healthy donor undergoing plastic surgery, with the informed consent of the donors whose identities were kept strictly anonymous. Experimental design was conducted with respect to the ethical permissions, according to the LVMH recherche agreement DC-2025-7230. The principal requirements of the Declaration of Helsinki were used as the guidelines to protect the rights, safety, and well-being of the subjects participating in the study. Patients (49- and 58-year-old females) were informed in advance of the possible reuse of surgical residues for scientific purposes, in accordance with a clear information procedure that complied with the applicable institutional framework, and were given the opportunity to object. The samples were collected from the abdomen region, coded, and stored under conditions that ensured the confidentiality, traceability, and security of the associated data. Ethical guidelines (French Bioethics law of 2004) did not require the study to be reviewed or approved by an ethics committee because the samples were obtained from the surgical discard of anonymous healthy patients.

### 2.2. Isolation and Preparation of Human Dermal Fibroblasts (HDFs)

Following the removal of the hypodermal adipose tissue, a 0.7 mm-thick section of the reticular dermis layer was isolated using an electric dermatome (Zimmer Biomet, Warsaw, IN, USA). Dermal fragments were digested with collagenase I (2 h at 37 °C in a 2 mg/mL solution, Gibco^TM^, Fischer Scientific SAS, Illkirch, France). After mechanical homogenisation, human dermal fibroblasts (HDFs) were preferentially amplified through a single passage in DMEM medium (Gibco) supplemented with 1 g/L of D-glucose (Gibco), 10% fetal calf serum (Gibco), and 100 U/mL of penicillin–streptomycin (Gibco). They were then maintained in DMEM 1 g/L glucose (Lonza^TM^, Fisher Scientific SAS, Illkirch, France) supplemented with 10% of FBS (Gibco^TM^, Fischer Scientific SAS, Illkirch, France), 2 mM of L-Glutamine (Lonza), and 100 µg/mL of Primocin^®^ (InvivoGen, Toulouse, France). Human epidermal keratinocytes (HEK) were similarly obtained. All cultures were maintained at 37 °C under 5% of CO_2_.

### 2.3. Replication Senescence and Oxidative Stress Characteristics

To evaluate the impact of replicative senescence on the number of VDAC1/IP3R complexes per cell, we used young HDFs (passage 5 to 7) and HDFs aged through replicative senescence (passage 30 or 31). Oxidative stress was triggered by exposing HDFs to a culture medium supplemented with tert-butyl hydroperoxide (t-BHP) for 10 or 30 min. Immediately after t-BHP exposure, HDFs were rinsed and either immediately analyzed as described below or incubated for 24 h in control medium prior to assessment.

### 2.4. Gastrodia elata Orchid Extract Preparation and Characterization

The plant (Hyundai Bioland, Seoul, Republic of Korea) underwent a washing and drying process. The extraction phase involved the use of 40% ethanol as a solvent, conducted at room temperature over a period of four days. Following extraction, the resulting solution is subjected to filtration, effectively removing any solid particles and impurities. The filtrate is then concentrated through vacuum evaporation. The concentrate is subsequently dissolved in a 50% butylene glycol solution. This is followed by microfiltration to further refine the solution. *Gastrodia elata* is a highly valued herb in traditional Chinese medicine, recognized for its anticonvulsant, analgesic, sedative, hypnotic, nootropic, and anti-ageing effects on the central nervous system. Additionally, it was reported to enhance myocardial energy metabolism, exert anti-inflammatory effects, and boost immune function. This plant contains a variety of bioactive compounds, including gastrodin, 4-hydroxybenzyl alcohol, 4-hydroxybenzaldehyde, benzyl alcohol, 4-hydroxy-3-methoxybenzaldehyde, 4-hydroxy-3-methoxybenzyl alcohol, parishin, and parishin B and C. Among these, gastrodin is the principal active constituent. Gastrodin has demonstrated protective effects on cardiomyocytes against oxidative injury by promoting the nuclear translocation of Nrf2, regulating mitochondrial dynamics, and preserving mitochondrial structure and function. Based on these properties, we have selected *Gastrodia elata* as a candidate for protecting mitochondria-associated membrane integrity and mitochondrial function.

### 2.5. Proteasome Activity Assay

Peptidase activity of the proteasome was assayed using the fluorogenic peptide substrate succinyl-Leu-Leu-Val-Tyr-7-amido-4-methylcoumarin (LLVY-AMC; S6510, Sigma-Aldrich, Saint-Quentin-Fallavier, France) as previously described [31]. Samples were incubated with LLVY-AMC at 37 °C in assay buffer, and fluorescence resulting from AMC release was measured continuously (excitation of 360 nm, emission of 460 nm) using a fluorescence plate reader (SPARK^®^ Multimode Microplate Reader, Tecan, Männedorf, Switzerland). Chymotrypsin-like proteasome activity was calculated from the linear portion of the fluorescence curve and normalized to total protein content.

### 2.6. RT-qPCR

Total RNA was isolated from HDFs using the Nucleospin 96 RNA extraction kit (Macherey-Nagel SARL, Hoerdt, France), then qualified and quantified with DropSense96 (Unchained Labs, Particular Sciences Ltd., Dublin, Ireland). For RT-PCR analysis, equal amounts of total RNA were used as a template for cDNA synthesis using the High Capacity cDNA Reverse Transcription Kit (Applied Biosystems, ThermoFischer Scientific, Fisher Scientific SAS, Illkirch, France). The *Cyclin-dependent kinase inhibitor 1A* (*CDKN1A*/*p21*, Hs01121172_m1) and *β2-microglobulin* (*β2M*, Hs99999907_m1) PCR primers were from Applied Biosystems, specifically designed for the TaqMan technology. Real-time PCR was performed using the QuantStudio 7 Flex PCR system (Applied Biosystems). The primer’s efficiency was determined with a standard curve using cDNA from in vitro culture of fibroblasts. *CDKN1A* mRNA levels were calculated using the comparative Ct (Cycle Threshold) method, with the Ct being defined as the number of cycles required for the fluorescent signal to cross the threshold. The *β2M* gene was used as the housekeeping gene. After calculating the delta Ct (*CtCDKN1A*-*Ctβ2M*), results are expressed as a relative quantification (2-DCt), indicated as Arbitrary Units (AU).

### 2.7. Assessment of the Number of VDAC1/IP3R Complexes per Cell

The proximity between the mitochondrial voltage-dependent anion channel 1 (VDAC1) and the inositol 1,4,5-trisphosphate receptor (IP3R) located in the ER membrane was quantified using an immunohistochemistry-based proximity ligation assay (PLA) kit (NaveniFlex^TM^ Cell Red, Navinci, Uppsala, Sweden), as previously described and thoroughly validated against transmission electron microscopy [22,23,24,25]. First, cells were fixed in 10% formaldehyde (10 min) at room temperature and rinsed twice in PBS. After permeabilization (Triton 0.01% for 15 min), cells were incubated overnight with antibodies targeting VDAC1 (dilution 1/4000, ab14734, Abcam Limited, Cambridge, UK) and IP3R (dilution 1/8000, ab5804, Abcam Limited, Cambridge, UK). Subsequent PLA reactions were carried out following the manufacturer’s protocol and included nuclei counterstaining with Hoechst 33342 or a mounting medium with DAPI (H1399, ThermoFisher Scientific, Fischer Scientific SAS, Illkirch, France).

Fluorescence spots were captured using a Zeiss inverted fluorescence microscope (Axio Observer 7, Karl Zeiss SAS, Rueil-Malmaison, France) with a ×40 or ×63 objective lens and the AxioVision software (v4.9, Karl Zeiss SAS, Rueil-Malmaison, France). The number of fluorescent spots was quantified using the BlobFinder software (v3.2, Centre for Image Analysis, Uppsala University, Uppsala, Sweden). Results are expressed as the number of spots per nucleus, with 10 to 20 images analyzed per condition, each being performed in 3 to 6 biological replicates.

### 2.8. Cellular Reactive Oxygen Species (ROS) Quantification

CellROX^TM^ deep red (C10422, Invitrogen, ThermoFisher Scientific, Fischer Scientific SAS, Illkirch, France) was used as an indicator of cellular ROS. FBHs were incubated with CellROX^TM^ (5 µM) at 37 °C for 30 min. The nuclear indicator Hoescht 33342 (10 µg/mL) was used as a counterstain. After thorough rinsing with PBS, cellular ROS levels were immediately quantified using a Zeiss inverted fluorescence microscope (Axio Observer 7, Zeiss, Germany) at the excitation and emission pair of 644/665 nm at a fixed shutter speed for each series. Signal intensity per cell was quantified using the ImageJ^®^ Software v1.54.

### 2.9. Atomic Force Microscopy Analyses

Exploration was performed on cells labelled using the in situ PLA to visualize and localize the VDAC1/IP3R contacts at the cellular level. Following PLA labelling, cells were maintained in phosphate-buffered saline (PBS) and immediately subjected to atomic force microscopy (AFM) measurements in liquid conditions.

Instrumentation: Stiffness measurements were performed using a Resolve BioScope AFM (Bruker Nano Surface, Santa Barbara, CA, USA) coupled to an inverted fluorescence microscope (DMi8, Leica Microsystems, Wetzlar, Germany). The optical system was equipped with ×20 and ×40 air objectives and a ×63 oil-immersion objective (Leica, Germany), enabling simultaneous acquisition of fluorescence and mechanical maps. AFM data were acquired using Nanoscope software version 9.1, allowing correlative optical and mechanical imaging. The integrated MIRO View (Bruker Nano Surface, Santa Barbara, CA, USA) software enabled precise spatial correlation between AFM scans and optical images, allowing accurate superimposition of mechanical/topographical maps with fluorescence data.

AFM Data Acquisition: Force–indentation experiments were conducted using pyramidal-tip cantilevers with a nominal spring constant of 0.7 N/m. According to the manufacturer’s specifications, the tip radius ranged between 40 and 50 nm. Each cantilever was calibrated prior to measurements using the thermal tune method, and the deflection sensitivity was determined in contact mode on a sapphire substrate.

Immediately prior to AFM acquisition, glass-bottomed dishes containing in vitro cultured cells were placed onto the motorized XY stage of the AFM system. Measurements were performed in liquid using fluid PeakForce QNM/MiRO QNM mode by recording force–volume maps consisting of 40 × 40 force curves (1600 curves per map).

Two mapping configurations were used:

- VDAC1/IP3R complex-targeted maps: scan area of 9 µm^2^.
- Global cell maps: scan area of 100 µm^2^.

Force–volume maps were acquired at a rate of 7 Hz, using 2048 points per force curve, ensuring high-resolution indentation profiles.

Mechanical Data Analysis: Elastic modulus values were extracted by fitting the indentation portion of the force curves using the Sneddon contact mechanics model, which is appropriate for indentation by a rigid pyramidal indenter:

$$F=\frac{2E}{\pi (1-\nu^{2})}tan(\alpha )\;\delta^{2}$$

where F is the applied force, E is the Young’s modulus, ν is Poisson’s ratio, α is the half-angle of the pyramidal tip, and δ is the indentation depth. A Poisson’s ratio (ν) of 0.3 was assumed for all measurements.

Force–volume datasets were first processed using Nanoscope Analysis 3.0, then exported and further analyzed using BioMeca analysis software (version 1.3.7). Although the Sneddon model is derived for the indentation of a flat elastic half-space, it was considered appropriate for this study given the comparative design of the analysis. In addition, indentation depths were limited to less than one-third of the local sample thickness, reducing substrate influence and ensuring that measurements primarily reflected the mechanical properties of the targeted cellular structures.

All results were expressed in terms of Young’s modulus, which represents a realistic approximation of the effective local stiffness of the sample. Because all samples were processed identically, the results were primarily interpreted in relative terms.

Global Cell Analysis: Large-area scans were analyzed using indentation depths of approximately 0.5 µm, enabling integration of mechanical contributions from both the cytoplasm and underlying cytoskeletal structures.

VDAC1/IP3R complex-Specific Analysis: VDAC1/IP3R complexes were identified using the in situ PLA fluorescence labelling. This fluorescence signal was used to localize the AFM probe to the labelled regions. Mechanical measurements were then specifically acquired and selected from areas corresponding to the PLA-positive signals on high-resolution 9 µm^2^ maps, using a tomography-based approach (as described in Roduit et al. [32]) to segment force–indentation curves into multiple depth layers. This allowed the extraction of mechanical properties from a defined indentation range corresponding to a specific subcellular layer.

### 2.10. Generalized Polarization (GP) Measurement

Membrane lipid order was assessed using the generalized polarization (GP) index measured with the solvatochromic fluorescent probe Laurdan (6-dodecanoyl-2-dimethylaminonaphthalene, Thermo Fisher Scientific, D250). The GP index was calculated according to the following equation:

$$\mathrm{GP}\;=\;\frac{I440\;-\;I490}{I440\;+\;I490}$$

where I440 and I490 correspond to the fluorescence emission intensities measured at 440 nm and 490 nm, respectively, upon excitation at ~350 nm. GP values range from −1, indicative of highly fluid and disordered membranes, to +1, corresponding to highly ordered membrane environments.

The samples were the same as those used for the AFM analyses described above. Cells were incubated with Laurdan, followed by washing steps prior to fluorescence acquisition. Emission spectra or images were acquired using a Zeiss microscope equipped with a 63× oil-immersion C-Apochromat objective (Karl Zeiss SAS, Rueil-Malmaison, France). Raw data were corrected for background signal and autofluorescence before GP calculation. Image analyses were performed using ImageJ and Python 3.12. The GP was calculated pixel by pixel in the image and per focal plane, and then an average value was extracted.

### 2.11. Mitochondrial Calcium Quantification

Living fibroblasts were loaded with the calcium-sensitive dye Rhod-2 using its cell-permeable Rhod-2 acetoxymethyl ester form (Rhod-2 AM, Thermofisher Scientific, R1244) dissolved at 4 µmol/L in the culture medium. After incubation (30 min at 37 °C, 5% CO_2_) and thorough rinsing with PBS, calcium levels were immediately quantified using a Zeiss inverted fluorescence microscope (Axio Observer 7, Zeiss, Germany) at the excitation and emission pair of 557/581 nm at a fixed shutter speed for each series. Signal intensity per mitochondrion was quantified using the ImageJ^®^ Software and the protocol published by J. Ross [33], with a minimum of 10 images per condition, each assessed in 3 biological replicates.

### 2.12. Evaluation of Mitochondrial Function

**Figure A1:**
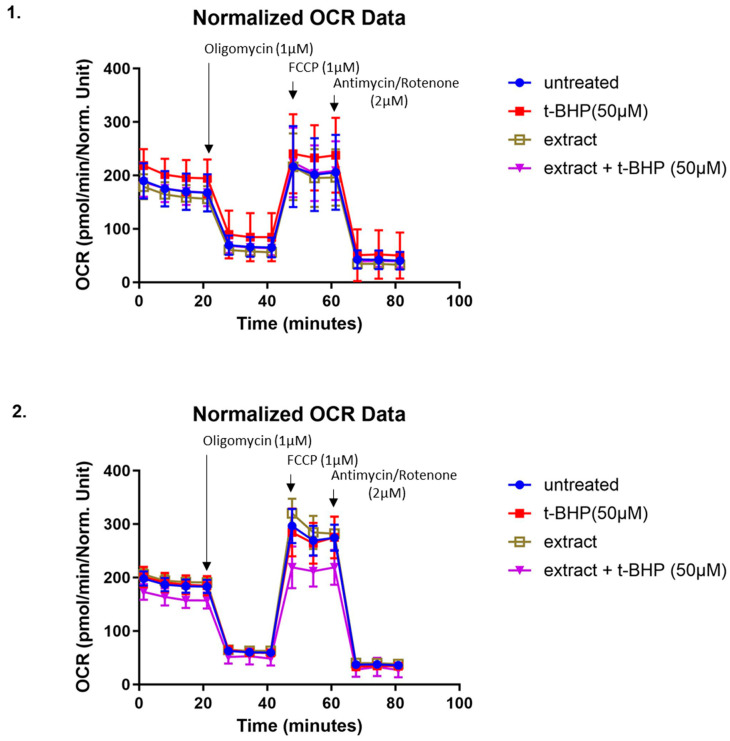
(**1**) After acute t-BHP treatment (50 µM) for 30 min, and (**2**) 24 h after acute t-BHP treatment.

Mitochondrial respiration was evaluated using a Seahorse XF96 (Agilent Technologies, Santa Clara, CA, USA) extracellular flux analyser. A total of 4 × 10^4^ cells was plated on an XF96 cell-culture microplate. On the following day, the medium was substituted with XF assay medium, and sequential injections of 1 μM oligomycin A, 1 μM FCCP, and 2 μM antimycin/rotenone were administered (Appendix A.1 Figure A1). Measurements were normalized by direct imaging of the cells via Hoescht-stained nuclei. Every condition was assessed in 8 technological replicates. Calculations were performed according to the Agilent Seahorse XF Cell Mito Stress Test Kit User Guide. Bioenergetic Health Index (BHI) was calculated using the following equation [34]:

$$\mathrm{BHI}\;=\;\frac{\left(\mathrm{Reserve}\;\mathrm{capacity})\;\times \;(\mathrm{ATP}\text{-}\mathrm{linked}\;\mathrm{respiration}\right)}{\;\left(Non\text{-}mitochondrial\;respiration\right)\times (Proton\;leak)}$$

### 2.13. Calculation of the MAM Flexibility Index (Iflex)

We hypothesized that cellular bioenergetic health depends on both the acute and long-term adaptation of calcium flux between the ER and mitochondria. To test this hypothesis, we developed a flexibility index (I*flex*) that captures both the acute response of VDAC1/IP3R complex abundance to a stress (e.g., 30 min t-BHP-induced oxidative stress) and the long-term adaptation after stress removal (e.g., 24 h recovery in control medium). The calculation of I*flex* was inspired by studies of metabolic flexibility in humans [35,36]. The number of VDAC1/IP3R complexes per nucleus measured by in situ PLA immediately after stress (Ni_0_) and 24 h after recovery (Ni_24h_) was first normalized to the mean of the corresponding control replicates, as follows:

If Ni/Mean(Ncontrol) ≥ 1:

$$\mathrm{xNi}\;=\;\frac{\mathrm{Ni}}{\mathrm{Mean}\left(\mathrm{Ncontrol}\right)\;}-1$$

If Ni/Mean(Ncontrol) < 1:

$$\mathrm{xNi}\;=\;1-\frac{\mathrm{Mean}(\mathrm{Ncontrol})}{\mathrm{Ni}}$$

We then calculated three components, as described below. ∆stress reflects the acute change in the number of VDAC1/IP3R complexes in response to stress relative to untreated controls. ∆correction represents the long-term adaptation after stress, assessing whether the number of complexes returns to control levels 24 h after stress removal. ∆reversion captures the difference between the acute and long-term responses of VDAC1/IP3R complexes. The closer ∆reversion is to zero, the lower the flexibility of the VDAC1/IP3R complexes:

$$∆\mathrm{stress}\;=\;{\mathrm{xNi}}_{0}\;-{\;\mathrm{Mean}(\mathrm{xNcontrol}}_{0})$$

$$∆\mathrm{correction}={\mathrm{xNi}}_{24h}-{\;\mathrm{Mean}(\mathrm{xNcontrol}}_{24h})$$

$$∆\mathrm{reversion}=\mathrm{abs}({\mathrm{xNi}}_{24h}-{\;\mathrm{xNi}}_{0})$$

For all conditions, I*flex* was calculated as follows:

$$I\mathrm{flex}\;=\;\frac{∆\mathrm{correction}\;\times \;∆\mathrm{stress}}{(1\;+\;∆\mathrm{reversion})}$$

Therefore, I*flex* approaches 0 when a treatment has little effect on the number of VDAC1/IP3R complexes and/or when this number returns close to control levels after 24 h, as observed for the control condition. In contrast, I*flex* increases as treatments cause larger changes in the number of VDAC1/IP3R complexes and/or prevent their return to control values after 24 h.

### 2.14. Statistical Analysis

Data were analyzed using the Graphpad Prism 10 software (GraphPad Software, San Diego, CA, USA). Results are presented as the mean ± standard deviation (SD). Data distribution was assessed using the Shapiro–Wilk test (α < 0.1). Normally distributed data were compared using two-way ANOVA, followed by Tukey’s multiple comparisons test. Other comparisons were conducted using the non-parametric Mann–Whitney test. The Pearson correlation coefficient was derived from simple linear regression. When necessary, correlation was explored using centred second-order polynomial (quadratic) regression.

## 3. Results

### 3.1. Effects of Replicative Senescence on VDAC1/IP3R Complexes

**Figure 1:**
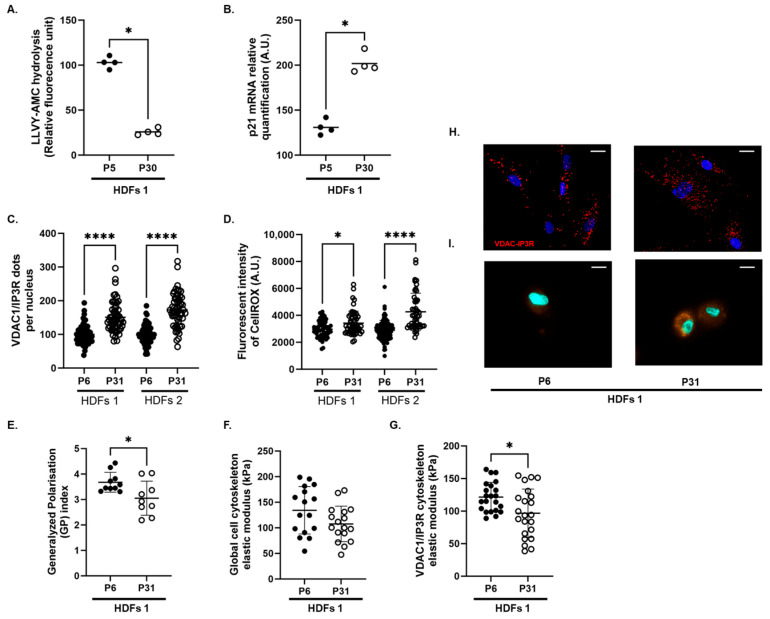
Effects of replicative senescence on (**A**) the proteasome activity assessed through the rate of hydrolysis of succinyl-Leu-Leu-Val-Tyr-7-Amido-4-Methylcoumarin (LLVY-AMC), (**B**) gene expression of p21, (**C**) the number of VDAC1/IP3R complexes per nucleus quantified using in situ proximity ligation assay, and (**D**) the cellular level of reactive oxygen species (ROS) assessed following CellROX^TM^ staining (n = 3 to 4 series). Exploration of the effects of replicative senescence on (**E**) the generalized polarization (GP) index of membrane fluidity, (**F**) the global elastic modulus of the cell cytoskeleton resistance, and (**G**) the elastic modulus of the cytoskeleton resistance in the vicinity of the VDAC1/IP3R complexes. Panels (**E**–**G**) show data obtained from a single experimental set. Results are presented for young human dermal fibroblasts (HDFs, P5 or P6) and HDFs aged through replicative senescence (P30 or P31) from two patients (HFDs1 and HFDs2). (**H**) Representative images of in situ proximity ligation assay between VDAC1 and IP3R (red dots). (**I**) Representative images of in-CellROX^TM^ staining (original magnification ×63 and scale bar = 20 µm). Statistical differences were analyzed using Mann–Whitney tests and are presented with *: *p* < 0.05, and ****: *p* < 0.0001.

Recent evidence unravelled that calcium fluxes from the ER to the mitochondria through the complex VDAC1/IP3R2 are drivers of senescence in human cells [17]. To assess the effect of replicative senescence on VDAC1/IP3R complexes, we used young HDFs (passages 5 and 6, P5 and P6) and replicatively senescent HDFs (passages 30 and 31, P30 and P31). Senescence markers were confirmed by decreased proteasome activity (Figure 1A) and increased *p21* mRNA levels (Figure 1B). In situ proximity ligation assay (PLA) was used to quantify the number of VDAC1/IP3R complexes per nucleus. The close proximity of VDAC1 and IP3R was assumed to reflect a functional complex involved in calcium transfer from the ER to mitochondria [24,37,38]. In the two different lines of HFDs analyzed, results from in situ PLAs indicated that replicative senescence increased the number of VDAC1-IP3R complexes per nucleus (Figure 1C, +85%, *p* < 0.001 in cell line 1; +72%, *p* = 0.007 in cell line 2). Interestingly, the assessment of cellular ROS level using the CellROX^TM^ probe assays indicated that replicative senescence enhanced the level of ROS (Figure 1D, +47%, *p* < 0.05 in cell line 1; +74%, *p* < 0.0001 in cell line 2).

We then examined whether a change in the number of VDAC1/IP3R complexes associated with replicative senescence was linked to alterations in cell membrane fluidity and the physical properties of the cytoskeleton, using atomic force microscopy in P6 and P31 of cell line 1. The generalized polarization (GP) index, which measured membrane fluidity, was 33.8% lower in P31 compared to P6 (*p* < 0.05) (Figure 1G), which means that replicative senescence was associated with a less fluid cell membrane. The cell elastic modulus, which measures the resistance of the cytoskeleton and thus its stiffness [39], was not significantly altered between P6 and P31 (Figure 1H). In contrast, the resistance of the cytoskeleton measured at the vicinity of the VDAC1/IP3R complexes (highlighted by the in situ PLA dots) was reduced by 20.4% in P31 compared to P6 HDFs (*p* < 0.05) (Figure 1I).

Oxidative stress is a significant contributing factor to replicative senescence in vitro [27,28]. Notably, hydrogen peroxide-induced oxidative stress in vitro has been shown to increase MAMs and the number of VDAC1/IP3R complexes [40], and also to reduce membrane fluidity [41]. These alterations closely mirror the adaptations observed in replicatively senescent HDFs compared with young HDFs, suggesting a central role for oxidative stress in this process. Based on these observations, we aimed to establish an acute oxidative stress model that recapitulates key features of replication-induced senescence and enables efficient biomolecule screening.

### 3.2. Building a Model of Acute Oxidative Stress

Tert-butyl hydroperoxide (t-BHP) is an organic hydroperoxide commonly used to induce oxidative stress in cell culture. Upon cellular metabolism, t-BHP generates ROS, including peroxyl and alkoxyl radicals, leading to glutathione depletion and oxidative damage [42]. Compared with hydrogen peroxide, t-BHP often induces a more sustained and reproducible oxidative stress [43], making it a widely used model in in vitro studies.

**Figure 2:**
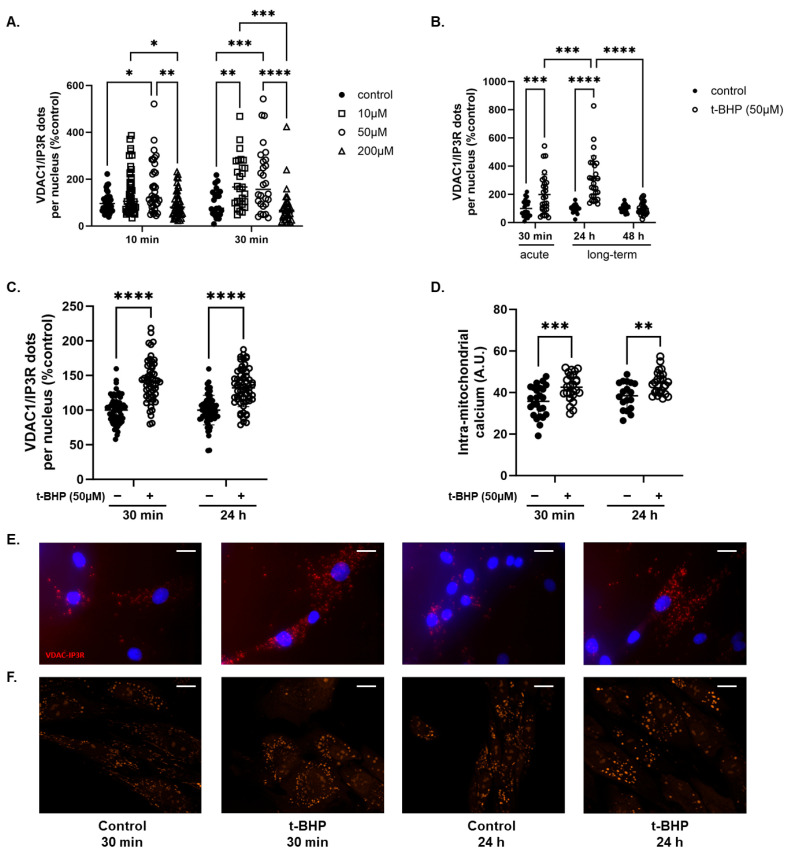
(**A**) Dose–response analysis of VDAC1/IP3R complexes quantified using in situ proximity ligation assays, after acute 10 and 30 min exposure to tert-butyl hydroperoxide (t-BHP) in human dermal fibroblasts (HDFs, passage 6). (**B**) Persistence of VDAC1/IP3R complex modulation at 24 and 48 h after a single acute 30 min exposure to t-BHP (50 µM) in HDFs (passage 6) (n = 2 series). Validation study: HDFs (passage 6 or 7) were exposed to t-BHP (50 µM) for 30 min. (**C**) VDAC1–IP3R complex formation (n = 6 series) and (**D**) intra-mitochondrial calcium levels assessed by Rhod-2 AM labelling (n = 3 series) were measured immediately after exposure (30 min) and 24 h after the acute oxidative stress. (**E**) Representative images of in situ proximity ligation assay between VDAC1 and IP3R (red dots) (original magnification ×63 and scale bar = 20 µm). (**F**) Representative images of Rhod2 AM staining (original magnification ×63 and scale bar = 20 µm). Statistical differences were analyzed using two-way ANOVA followed by Tukey’s multiple comparisons test. They are presented with, *: *p* < 0.05, **: *p* < 0.01, ***: *p* < 0.001, and ****: *p* < 0.0001.

We first studied the dose–response relationship of VDAC1/IP3R complex adaptation in HDFs (passage 6) to acute exposure (10 and 30 min) to 10, 50, and 200 µM t-BHP. After 10 min incubation, 50 µM t-BHP increased the number of VDAC1/IP3R complexes per cell by 64.6% compared to untreated cells (*p* < 0.05) (Figure 2A), whereas both 10 and 50 µM t-BHP doubled the number of VDAC1/IP3R complexes per cell after 30 min of exposure to t-BHP (*p* < 0.05) (Figure 2A). In contrast, both after 10 and 30 min incubation, 200 µM t-BHP decreased the number of VDAC1/IP3R complexes compared to the 50 µM condition (*p* < 0.05), returning it to the control level (Figure 2A). Using the most effective condition (50 µM t-BHP for 30 min) in agreement with Wedel et al. [29], we then sought to determine whether such acute oxidative stress had an impact on the number of VDAC1/IP3R complexes per cell after 24 and 48 h recovery. HDFs subjected to acute stress showed three times more VDAC1-IP3R complexes per cell than untreated HDFs after 24 h recovery (*p* < 0.0001), but this difference was no longer visible after 48 h (Figure 2B). These results indicate that the impact of acute oxidative stress extends over 24 h, supporting a simplified yet effective model of senescence-like behaviour.

### 3.3. Validation of the Acute Oxidative Stress Model

After confirmation of the impact of acute oxidative stress on the VDAC1/IP3R complexes (Figure 2C), we explored the consequences on intra-mitochondrial calcium levels (Figure 2D). Compared to untreated controls, a 30 min treatment with 50 µM t-BHP increased mitochondrial calcium levels by 39% (*p* < 0.0001). After 24 h, this increase reached 52% (*p* < 0.0001), even though HDFs had been returned to the control medium.

Calcium uptake into the mitochondrial matrix is critically important for mitochondrial function [44]. We thus evaluated several parameters of mitochondrial respiration, focusing on the resulting Bioenergetic Health Index (BHI). BHI is a quantitative indicator of mitochondrial function and cellular energy status. A higher BHI reflects better mitochondrial performance and greater cellular resilience to stress [34].

In acute experiments, although the difference did not reach the level of significance, HDFs exposed to oxidative stress exhibited a reduction in the BHI compared with untreated cells (−40%; Table 1), reflecting an impairment of cellular bioenergetics. This was associated with elevated basal (+14%, *p* < 0.05) and maximal (+12%, *p* < 0.05) respiration rates and non-mitochondrial respiration (+22%, *p* < 0.001). In contrast, ATP-linked respiration and reserve capacity were not significantly affected (Table 1).

Twenty-four hours after acute oxidative stress, the BHI remained significantly reduced in t-BHP-exposed HDFs compared with untreated cells (−27%, *p* < 0.05; Table 2), indicating a persistent impairment of cellular bioenergetics. In contrast to the acute response, the decrease in BHI was primarily associated with a reduction in reserve capacity (−17%, *p* < 0.01). Other mitochondrial and respiratory parameters were not significantly altered in comparison to untreated HDFs (Table 2).

**Figure 3:**
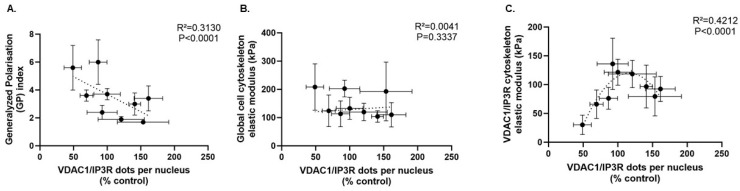
Correlations between the number of VDAC1/IP3R complexes per nucleus and (**A**) the generalized polarization (GP) index of membrane fluidity, (**B**) the global elastic modulus of the cell cytoskeleton resistance, and (**C**) the elastic modulus of the cytoskeleton resistance in the vicinity of the VDAC1/IP3R complexes. Measurements were performed on a single experimental set after 24 h in untreated and treated (50 µM t-BHP for 30 min with or without pre- and post-treatment with orchid extract) HDFs (passage 6). Correlations were explored by simple linear regression and centred second-order polynomial (quadratic) regression.

Finally, we also studied the impact of acute oxidative stress on membrane fluidity and the physical properties of the cytoskeleton, assessed using an atomic force microscope, and compared HDFs subjected to acute oxidative stress with untreated control HDFs after 24 h. Impact of a *Gastrodia elata* orchid extract under both conditions was also included. Interestingly, the generalized polarization (GP) index was negatively correlated with the number of VDAC1/IP3R complexes per cell (R^2^ = 0.313, *p* < 0.0001) (Figure 3A), meaning that the more complexes there are, the less fluid the cell membrane is. In contrast, the cell elastic modulus was not correlated with the number of VDAC1/IP3R complexes per cell (Figure 3B). Yet, when similar measurements were taken in the vicinity of the VDAC1/IP3R complexes (highlighted by the in situ PLA dots), cytoskeletal rigidity followed a U-shaped curve, with a maximum in untreated control HDFs, and a decrease when the number of VDAC1/IP3R complexes per cell either increased or decreased (Figure 3C).

### 3.4. Effects of Oxidative Stress on a Flexibility Index (Iflex)

We hypothesized that 24 h BHI depends on both acute and long-term mitochondrial adaptation to changes in calcium flux between the ER and mitochondria. To test this hypothesis, we developed a flexibility index (Iflex) that captures both the acute response of VDAC1/IP3R complex abundance to a stress (e.g., 30 min t-BHP-induced oxidative stress) and the long-term adaptation after stress removal (i.e., 24 h recovery in control medium). The rationale for developing this index was to quantify the ability of cells to adapt to and recover from stressors such as oxidative stress. The Iflex index, inspired by studies of metabolic flexibility in humans [35,36], was calculated using in situ PLA data obtained immediately and 24 h after t-BHP stress vs. control (see Section 2).

**Figure 4:**
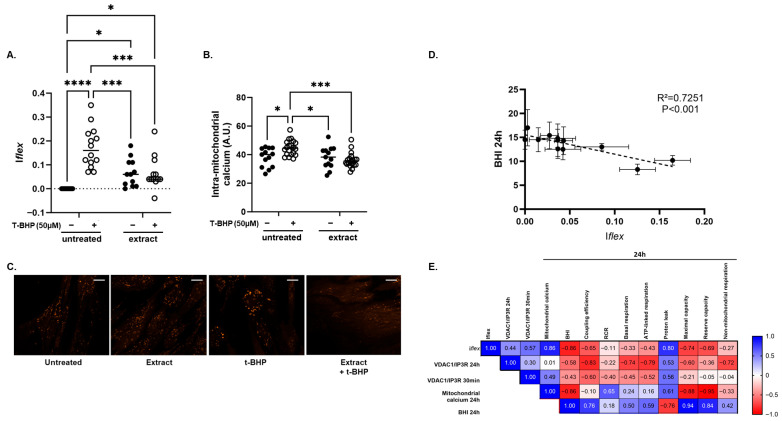
Effects of acute oxidative stress on (**A**) the VDAC1/IP3R flexibility index (Iflex) and (**B**) the intra-mitochondrial calcium content assessed following Rhod2 AM staining, in human dermal fibroblasts (HDFs, passage 6 or 7) (n = 3 series). (**C**) Representative images of Rhod2 AM staining (original magnification ×63 and scale bar = 20 µm). The HFDs were treated with 0.05% orchid extract (Gastrodia elata) for 40 h, 16 h before and 24 h after t-BHP treatment (50 µM, 30 min). Statistical difference was analyzed using two-way ANOVA followed by Tukey’s multiple comparisons test. It is presented with *: *p* < 0.05, ***: *p* < 0.001, and ****: *p* < 0.0001. (**D**) Linear regression between Iflex and BHI measured at 24 h (BHI 24 h) in HDFs (passage 6 or 7). (**E**) Matrix of correlation (Pearson r) between Iflex, the number of VDAC1/IP3R complexes measured at 30 min (VDAC1/IP3R 30 min) and 24 h (VDAC1/IP3R 24 h), BHI measured at 24 h (BHI 24 h), and mitochondrial respiration parameters measured at 24 h in HFDs (passage 6 or 7).

In untreated HDFs, the Iflex equaled zero, indicating stability in the number of VDAC1/IP3R complexes per cell between measurements (30 min, 24 h) (Figure 4A). In contrast, HDFs subjected to acute t-BHP stress exhibited a significant increase in Iflex (0.119 ± 0.073, *p* < 0.0001), reflecting acute and long-term alterations in VDAC1/IP3R complexes compared to untreated HFDs (Figure 4A).

**Figure A2:**
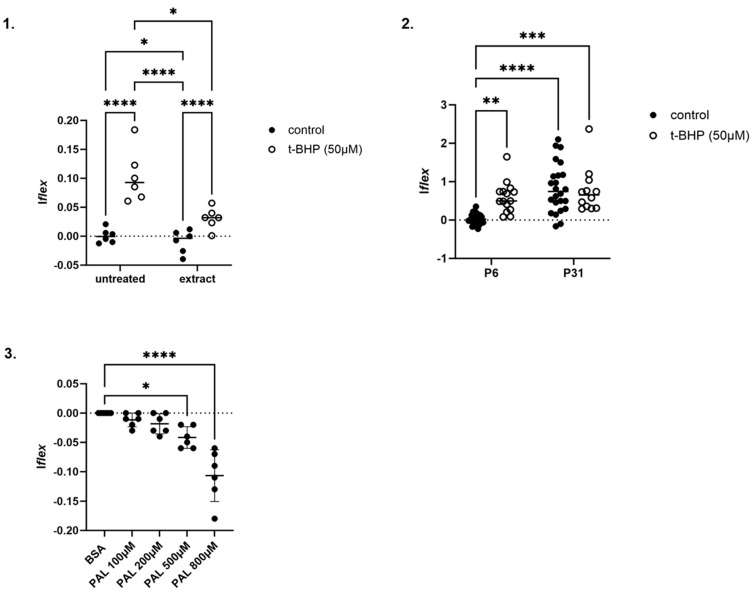
IP3R flexibility index (Iflex) in (**1**) human epidermal keratinocytes in basal conditions and following treatment with 0.05% orchid extract (Gastrodia elata) (passage 5); (**2**) young human dermal fibroblasts (passage 6, P6) and replicatively senescent human dermal fibroblasts (passage 31, P31). Statistical difference was analyzed using two-way ANOVA followed by Tukey’s multiple comparisons test, and is presented with *: *p* < 0.05, ** *p* < 0.01, ***: *p* < 0.001 and ****: *p* < 0.0001. (**3**) Effect of palmitate-induced lipotoxicity for 3 h on the VDAC1/IP3R flexibility index (I*flex*) in primary human fibroblasts (passage 6), showing that 500 µM and 800 µM palmitate for 3 h remain lipotoxic 24 h after returning to a control medium. Statistical difference was analyzed using the Kruskal–Wallis test, and is presented with *: *p* < 0.05, and ****: *p* < 0.0001. Measurements were performed on a single set, with 6 technical replicates.

The impact of the Gastrodia elata orchid extract was evaluated under both conditions. Compared to untreated HDFs, the orchid extract slightly increased Iflex in control conditions (0.067 ± 0.057, *p* < 0.05). Upon oxidative stress, it maintained Iflex at levels similar to those of the unstressed control (0.068 ± 0.007, *p* = 0.999) and significantly lower than that of t-BHP-treated HDFs (*p* = 0.0004) (Figure 4A), evidencing that the orchid extract protected the VDAC1/IP3R complexes from oxidative stress-induced alterations. Interestingly, Iflex explored on keratinocytes using the same protocol evidenced that the orchid extract was less protective against t-BHP stress than in HDFs, as Iflex was only partially corrected compared to t-BHP-treated keratinocytes (*p* < 0.05) (Appendix A.2, Figure A2(1)). Furthermore, Iflex demonstrated a loss of adaptability to acute stress induced by t-BHP in replicative senescent HDF cells compared to young HDF cells (Appendix A.2 Figure A2(2)). Finally, and in other respects, Iflex was discriminant in exploring the long-term (24 h) impact of a 3 h-acute palmitate-induced lipotoxicity in HDFs (Appendix A.2 Figure A2(3)).

To corroborate these results, we quantified the intra-mitochondrial calcium levels under the same conditions (Figure 4B). The statistical analysis yielded identical trends: the orchid extract alone had no significant effect itself compared to untreated HDFs (*p* = 0.823) and prevented the t-BHP-induced increase in intra-mitochondrial calcium (*p* < 0.001), thus maintaining this parameter at control levels (*p* = 0.818).

Similarly, exploration of mitochondrial function showed that the orchid extract prevented the degradation of BHI both immediately after t-BHP stress and after 24 h recovery (Table 1 and Table 2). Supporting our hypothesis, Iflex measured in HDFs better correlated with BHI (Pearson r = −0.794, *p* = 0.004) (Figure 4D) and mitochondrial calcium (Pearson r = 0.758, *p* = 0.002) than the number of VDAC1/IP3R complexes per cell after 24 h (Pearson r = 0.228 and −0.523, *p* = 0.476 and 0.081, respectively) (Figure 4E). These observations support the notion that, in response to an acute stress, mitochondrial function adaptation depends on both acute and long-term mechanisms, which are based in particular on acute and long-term adaptation of communication between mitochondria and the ER.

## 4. Discussion

Whether intrinsic—genetically determined—or extrinsic—related to environmental stressors—ageing leads to skin decline [45]. While substantial progress has been made in elucidating the mechanisms underlying age-related extracellular matrix degradation and fibroblast senescence, the potential contribution of MAM alterations to fibroblast dysfunction in ageing remains largely unexplored. However, accumulating evidence suggests that changes in MAM integrity play a crucial role in both ageing and age-related diseases [10,13,15,16,17,46].

Using HDFs—the main dermal cell type responsible for synthesizing the extracellular matrix that provides structural support to the skin—we found that replicative senescence increases the number of VDAC1/IP3R complexes per cell, suggesting higher contact points between the ER and the mitochondria. The effects of ageing on MAM tethering appear to be context-dependent, varying according to the study, the cell type evaluated, and the model examined. While reports describe a reduction in MAM tethering with ageing in striated muscle [15], enhanced MAM contact sites have been documented in various senescent cell types (e.g., porcine aortic endothelial cells and MRC5, IMR90, and WI38 human fetal lung fibroblasts) [17,47,48,49]. Furthermore, oxidative stress, a signalling mediator and a source of cellular damage in both intrinsic and extrinsic ageing [50,51], also led to an increase in the number of VDAC1/IP3R complexes per cell within 30 min up to 24 h. At both time points, this increase was accompanied by higher intra-mitochondrial calcium levels, which is consistent with the fact that VDAC1/IP3R complexes are critical for controlling calcium transfer to mitochondria [52]. The rapid impact, within 10 to 30 min, of 50 µM t-BHP on VDAC1/IP3R complexes is consistent with a recent study that demonstrated that the *PERK-ERO1* module is a key molecular mechanism enabling rapid, redox-dependent adaptation of MAM function and mitochondrial metabolism in response to ROS [53]. Interestingly, the effect is not only rapid but also long-lasting. Although the level of residual ROS after 24 h of t-BHP treatment is difficult to assess, the number of VDAC1/IP3R complexes remained elevated at this later time point, with mitochondrial calcium accumulation still pronounced. This raises the possibility of a self-perpetuating mechanism that may evolve into a vicious circle. However, the dose is the issue. Indeed, our initial dose–response experiment showed that 200 µM of t-BHP over 30 min significantly reduced the number of VDAC1/IP3R complexes compared to the 50 µM dose, highlighting that different mechanisms are involved, depending on the extent of oxidative stress.

The increase in calcium transfer from the ER to the mitochondria associated with the increase in VDAC1/IP3R complexes contributes to greater intra-mitochondrial calcium accumulation [17,47,48,49]. This leads to acute stimulation of key calcium-dependent mitochondrial dehydrogenases of the Krebs cycle and enhanced mitochondrial respiration [54,55,56]. In addition, chronic accumulation of mitochondrial calcium may ultimately lead to increased mitochondrial ROS production, decreased mitochondrial respiration [57,58], and accelerated cellular senescence [59,60]. Overall, our results are consistent with these findings, stating that oxidative stress is sufficient to promote the formation of VDAC1/IP3R complexes, elevate intra-mitochondrial calcium levels, and impair mitochondrial function both acutely and at a longer term. However, in our model, acute oxidative stress did not alter mitochondrial ATP-linked respiration or coupling efficiency acutely and at 24 h, but instead affected the maximal respiration or the reserve capacity. Hence, it acutely enhanced the maximal respiration, but yielded to a decreased reserve capacity at 24 h, resulting in a 27% lower BHI. This means that over the long term, such a reduction in reserve respiratory capacity may limit mitochondrial adaptability, potentially predisposing cells to metabolic dysfunction under sustained or repeated stress conditions.

The *Gastrodia elata* extract, which is rich in gastrodin, has been reported to exert protective effects against oxidative injury, notably through the activation of the Nrf2 pathway, regulation of mitochondrial dynamics, and preservation of mitochondrial structure and function [30]. In line with these findings, our results demonstrate that the orchid extract at 0.05% is sufficient to mitigate mitochondrial dysfunction induced by acute t-BHP exposure. Although a reduction in ATP-linked respiration was observed at 24 h (−13%, *p* < 0.001), this effect was accompanied by a significant increase in the reserve capacity compared to control cells exposed to t-BHP (+28%, *p* < 0.05). This shift suggests a metabolic adaptation rather than a deleterious effect, indicating an improved mitochondrial response to energetic stress. Consistently, these changes were associated with the maintenance of BHI at levels comparable to untreated control cells, supporting a preservation of overall mitochondrial function following oxidative stress.

Replicative-induced senescence and t-BHP exposure were associated with higher plasma membrane rigidity. A direct relationship between ROS-induced lipid peroxidation and decreased membrane fluidity is likely [41]. This effect may also arise from alterations in cholesterol metabolism [61,62]. Higher plasma membrane cholesterol was found in replicative senescence [63]. In addition, previous studies in hepatocellular carcinoma cells have shown that reinforcement of MAM architecture through GRP75 or Mitofusin-2 overexpression causally modifies cholesterol flux and promotes intracellular cholesterol accumulation [64].

It is very interesting to note that our exploratory approach, which employed atomic force microscopy (AFM), showed that our acute oxidative stress model had not altered the overall rigidity of the cytoskeleton, but rather induced a localized adaptation of the cytoskeleton in the vicinity of MAM microdomains. Indeed, the higher number of VDAC1/IP3R complexes in replicative senescence and following t-BHP stress was associated with reduced cytoskeleton rigidity in the vicinity of the protein complexes. Oxidative stress was shown to modulate cytoskeletal dynamics through direct oxidative modifications of actin and tubulin residues, enzymatic regulation of localized ROS production affecting actin polymerization and filament severing, and by altering the stability and interaction of microtubules and their associated proteins [65,66]. More broadly, our data indicate for the first time that changes in MAM contact sites are associated with local changes in cytoskeletal rigidity, potentially due to alterations in its structure, tension, and function. It is not possible to determine whether the degradation or depolarization of the cytoskeleton captured by the atomic force microscopy is a consequence of oxidative stress or the intrinsic mechanisms necessary for the formation of MAM microdomains. This question requires further investigation.

Finally, using an in situ PLA approach, we developed the I*flex* index. The rationale behind I*flex* was to quantify in a single value the effect of acute and long-term adaptive responses of the VDAC1/IP3R complexes in response to stress and/or treatment. I*flex* intends to facilitate the screening and comparison of treatments that target mitochondrial function, as it considers both acute dynamics in response to stress and the ability to return to baseline conditions. Preliminary assessment of this index logically demonstrates that, when comparing an untreated control to itself, no differential effect is observed and the I*flex* remains close to zero. Conversely, a 30 min t-BHP treatment, which induces both acute and long-lasting changes in VDAC1/IP3R complexes, results in a marked increase in the I*flex* value. Consistent with the fact that I*flex* effectively integrates acute and chronic adaptations, I*flex* correlated better with mitochondrial calcium and BHI at 24 h than with separate measurements of VDAC1/IP3R complexes at 30 min and 24 h. This index, therefore, has potential for assessing the impact of various metabolic fluctuations or stress (e.g., t-BHP-induced oxidative stress and palmitate-induced lipotoxicity). It also aimed to quantify the efficacy of compounds that could mitigate MAM dynamic perturbations. The I*flex* value obtained after treatment with the orchid extract in both HDFs and HDKs suggests that the index could fulfil this objective. These initial results are encouraging and are supported by measurements of intra-mitochondrial calcium concentrations and BHI values. In light of this, the I*flex* index represents a useful and complementary approach to existing metrics. However, further studies are required to fully establish its relevance and robustness.

The in situ PLA was selected in the present study due to its relative efficiency, reduced acquisition time, and suitability for higher-throughput screening, enabling the analysis of multiple conditions in a consistent and comparative manner, under carefully controlled conditions to ensure reproducibility [26]. In situ PLA-based quantification of VDAC1/IP3R interactions thus provides a valuable and accessible proxy for MAM abundance and dynamics. However, it presents several limitations compared with ultrastructural approaches. Indeed, in situ PLA detects protein proximity rather than direct physical contacts, and therefore may overestimate functional MAMs by capturing indirect or transient associations. This is further constrained by the intrinsic proximity threshold of the assay (<40 nm), which, although offering relatively high resolution, reflects spatial closeness rather than bona fide functional molecular interaction. This may contribute to potential overinterpretation. In order to mitigate these inherent limitations, it is important to note that the findings from the PLA analysis were consistent with functional approaches, namely mitochondrial calcium concentration and mitochondrial respiration. The convergence of these independent measures supports the reliability of the conclusions drawn from the PLA analysis and reinforces their biological relevance.

## 5. Conclusions

Similar to the effects observed in replicative senescent primary HDFs, oxidative stress is sufficient to increase plasma membrane rigidity, VDAC1/IP3R complexes, and mitochondrial calcium levels, and impair mitochondrial maximal respiration and reserve capacity. This interplay is likely to establish a self-perpetuating cycle of mitochondrial dysfunction, possibly contributing to cellular senescence. Given that oxidative stress is a hallmark of the environmental insults faced by the skin, our findings suggest that its impact on MAM integrity in skin cells could play a significant role in extrinsic skin ageing.

## Figures and Tables

**Table 1:** Mitochondrial respiration parameters of untreated HDFs and HDFs treated acutely with 50 µM t-BHP, in basal conditions and following pretreatment with 0.05% Orchid extract (*Gastrodia elata*) for 16 h before t-BHP treatment (50 μM, 30 min).

Mitochondria Respiration Parameters (pmol/min)	Untreated Control	t-BHP Treated Control	Extract	Extract t-BHP Treated
Basal respiration	131.2 ± 5.4	149.3 ± 7.0 **	124.1 ± 7.3	130.9 ± 6.6 $ ###
ATP-linked respiration	106.9 ± 4.9	108.7 ± 6.1	101.6 ± 4.1 ##	106.8 ± 6.2
Maximal respiration	166.1 ± 6.3	186.0 ± 5.2 *	168.4 ± 18.1	171.6 ± 14.1
Proton leak	26.2 ± 2.7	35.4 ± 2.5 *	24.4 ± 5.4	29.8 ± 2.2 $
Reserve capacity	34.1 ± 7.3	34.9 ± 10.2	40.8 ± 10.0	39.1 ± 10.1
Non-mitochondrial respiration	41.8 ± 0.8	51.1 ± 1.1 ***	34.1 ± 1.6 ***	39.6 ± 3.6 $$$ ###
Coupling efficiency (%)	79.4 ± 2.4	75.7 ± 1.3	80.8 ± 3.0	79.3 ± 5.9
Respiratory control ratio (RCR)	3.09 ± 0.56	2.86 ± 0.44	3.69 ± 0.34 *	3.45 ± 0.51 #
BHI	3.7 ± 0.7	2.2 ± 0.4	4.9 ± 1.5 **	4.0 ± 0.8 $ ##

N = 3 series. Statistical differences were analyzed using two-way ANOVA followed by Uncorrected Fisher’s LSD test, compared to untreated control: *: *p* < 0.05, ** *p* < 0.01, and ***: *p* < 0.001; compared to untreated extract: $: *p* < 0.05, and $$$: *p* < 0.001, and compared to t-BHP treated control: #: *p* < 0.05, ##: *p* < 0.01, and ###: *p* < 0.001.

**Table 2:** Mitochondrial respiration parameters of untreated HDFs and HDFs treated acutely with 50 µM t-BHP after 24 h recovery, in basal conditions and following pre- and post-treatment with 0.05% orchid extract (*Gastrodia elata*) for 40 h, 16 h before and 24 h after t-BHP treatment (50 μM, 30 min).

Mitochondria Respiration Parameters (pmol/min)	Untreated Control	t-BHP Treated Control	Extract	Extract t-BHP Treated
Basal respiration	111.8 ± 4.4	111.7 ± 4.3	104.8 ± 4.8 **	99.7 ± 4.1 $$ ###
ATP-linked respiration	88.7 ± 4.4	87.0 ± 2.7	82.7 ± 3.3 **	77.8 ± 3.3 $$ ###
Maximal respiration	222.7 ± 22.6	197.2 ± 22.0	225.8 ± 28.6	207.7 ± 27.1
Proton leak	23.1 ± 1.8	24.9 ± 2.7	21.9 ± 2.6	21.7 ± 2.3 #
Reserve capacity	103.8 ± 18.1	86.0 ± 13.7 **	115.2 ± 13.2	110.5 ± 19.9 #
Non-mitochondrial respiration	20.8 ± 2.1	29.2 ± 2.0	30.3 ± 1.8	29.2 ± 2.2
Coupling efficiency (%)	79.3 ± 1.4	77.9 ± 1.4	79.0 ± 1.6	78.1 ± 1.7
Respiratory control ratio (RCR)	3.75 ± 0.32	3.83 ± 0.33	3.46 ± 0.31	3.54 ± 0.30
BHI	14.2 ± 1.6	10.3 ± 0.9 *	13.5 ± 2.2	12.7 ± 2.3 $

N = 3 series. Statistical differences were analyzed using two-way ANOVA followed by Uncorrected Fisher’s LSD test, compared to untreated control: *: *p* < 0.05, and **: *p* < 0.01; compared to untreated extract: $: *p* < 0.05, and $$: *p* < 0.01, and compared to t-BHP treated control: #: *p* < 0.05, and ###: *p* < 0.001.

## Data Availability

The data that support the findings of this study are available from the corresponding author upon reasonable request.

## Abbreviations

The following abbreviations are used in this manuscript:

AFM	Atomic force microscopy
BHI	Bioenergetic Health Index
ER	Endoplasmic reticulum
GP	Generalized polarization
HDF	Human dermal fibroblast
HEK	Human epidermal keratinocyte
Iflex	Flexibility index
In situ PLA	In situ proximity ligation assay
IP3R	Inositol 1,4,5-triphosphate receptor
MAM	Mitochondria–ER-associated membranes
MCS	Membrane contact site
MERC	Mitochondria–ER contact site
P	Passage
RCR	Respiratory control ratio
ROS	Reactive oxygen species
t-BHP	Tert-butyl hydroperoxide
TEM	Transmission electron microscope
VDAC1	Voltage-dependent anion channel 1
